# Co-design of the Transgender Health Information Resource: Web-Based Participatory Design

**DOI:** 10.2196/38078

**Published:** 2023-01-10

**Authors:** Brad Morse, Andrey Soares, Kate Ytell, Kristen DeSanto, Marvyn Allen, Brooke Dorsey Holliman, Rita S Lee, Bethany M Kwan, Lisa M Schilling

**Affiliations:** 1 Division of General Internal Medicine University of Colorado Anschutz Medical Campus Aurora, CO United States; 2 Data Science to Patient Value Initiative School of Medicine University of Colorado Anschutz Medical Campus Aurora, CO United States; 3 Elevance Health Denver, CO United States; 4 Strauss Health Sciences Library University of Colorado Anschutz Medical Campus Aurora, CO United States; 5 One Colorado Denver, CO United States; 6 Department of Family Medicine University of Colorado Anschutz Medical Campus Aurora, CO United States; 7 Adult and Child Center for Outcomes Research and Delivery Science University of Colorado Anschutz Medical Campus Aurora, CO United States; 8 Department of Emergency Medicine University of Colorado Anschutz Medical Campus Aurora, CO United States

**Keywords:** transgender, gender diverse, participatory design, web-based design, co-design, health information resource, smartphone, app, mobile phone

## Abstract

**Background:**

There is an urgent and unmet need for accessible and credible health information within the transgender and gender-diverse (TGD) community. Currently, TGD individuals often seek and must find relevant resources by vetting social media posts. A resource that provides accessible and credible health-related resources and content via a mobile phone app may have a positive impact on and support the TGD population.

**Objective:**

COVID-19 stay-at-home orders forced a shift in the methods used in participatory design. In this paper, we aimed to describe the web-based participatory methods used to develop the Transgender Health Information Resource. We also described and characterized the web-based engagement that occurred during a single session of the overall design process.

**Methods:**

We planned and conducted web-based design sessions to replace the proposed in-person sessions. We used web-based collaborative tools, including Zoom (Zoom Video Communications), Mural (Mural), REDCap (Research Electronic Data Capture; Vanderbilt University), and Justinmind (Justinmind), to engage the participants in the design process. Zoom was used as an integrated platform for design activities. Mural was used to perform exercises, such as free listing, brainstorming, and grouping. REDCap allowed us to collect survey responses. Justinmind was used to create prototypes that were shared and discussed via Zoom. Recruitment was led by one of our community partners, One Colorado, who used private Facebook groups in which web-based flyers were dispersed. The design process took place in several workshops over a period of 10 months. We described and characterized engagement during a single design session by tracking the number of influential interactions among participants. We defined an influential interaction as communication, either verbal or web-based content manipulation, that advanced the design process.

**Results:**

We presented data from a single design session that lasted 1 hour and 48 minutes and included 4 participants. During the session, there were 301 influential interactions, consisting of 79 verbal comments and 222 web-based content manipulations.

**Conclusions:**

Web-based participatory design can elicit input and decisions from participants to develop a health information resource, such as a mobile app user interface. Overall, participants were highly engaged. This approach maintained the benefits and fidelity of traditional in-person design sessions, mitigated deficits, and exploited the previously unconsidered benefits of web-based methods, such as enhancing the ability to participate for those who live far from academic institutions. The web-based approach to participatory design was an efficient and feasible methodological design approach.

## Introduction

### Transgender and Gender-Diverse Health and Medical Information Needs

Transgender and gender-diverse (TGD) individuals (defined as people whose current gender is different from that assigned at birth, including, but not limited to, nonbinary, queer, and gender nonconforming people, hereafter shortened to *TGD*) face health disparities including high degrees of stigma and discrimination from providers and health care systems [1-4]. Three-fourths of TGD individuals report negative experiences with the health care system [5]. TGD individuals report difficulties finding and accessing TGD-competent health care professionals [6-10], securing insurance coverage for their health care needs [11-13], and finding health care professionals who are sensitive to the needs of the TGD population [14]. Transgender individuals experience stigma and discrimination across the social determinants of health, including bullying in schools, lack of stable income, and quality housing [15-17]. Moreover, TGD individuals often have to manage chronic stress owing to traumatic experiences over their life course [18,19].

Owing to stigma and discrimination when seeking health care, TGD community members often turn to health and medical information on the web [20,21]. Documented examples of insensitive health care include gender insensitivity in which individuals were misgendered (using “he” when a “they” pronoun was requested) or forced care where some patients felt they were forced to do unnecessary examinations or dismissed as “psych cases” [22]. A growing body of literature focuses on the TGD community and their health information–seeking behavior on the web [20,23,24]. A study found that gender transition mental health message boards are popular, especially on the Tumblr platform [25]. In another study, younger TGD individuals used various web-based platforms to explore transgender, nonbinary, and gender-diverse identities and to find support networks [26]. There is evidence that social media is a key resource relied upon by the TGD community to obtain health and medical information [27]. The affordances of social media provide a network for peer-to-peer, emotional, appraisal, and informational support [27].

Considering the known difficulties in seeking and determining the credibility of web-based transgender health information and the current lack of transgender-specific materials on the web [20,24], we aimed to create a health information resource to support the TGD population. For this project, credible information was defined as information created or disseminated by clinicians and organizations, such as the Trevor Project [28], with expert knowledge about care specific to the needs of transgender individuals, and reputable sources of health information such as MEDLINEPlus [29]. Published literature highlights the lack of credible web-based information resources dedicated to the needs of TGD individuals [20,30,31]. Digital tools might be important for TGD health self-management, but they are currently underutilized [32].

To address this need, we developed the Transgender Health Information Resource (TGHIR). We chose a mobile app (Android and iOS mobile operating system) user interface as the optimal method of delivery because of mobile phone ownership—85% of Americans own a smartphone [33]—and mobile phones support anywhere and anytime access to information.

### Participatory Design and Web-Based Participatory Design

Participatory design has proven to be successful in designing mobile health resources. The benefits of this approach include engaging co-designers (henceforth, participants) selected to be representative of the community of intended end users to thoroughly explore and prioritize target audience needs [34-36]. Researchers and participants can collaborate and design [37] interfaces that make information accessible. Representation in the design process is helpful because researchers often do not understand how others are affected by technology performance [38].

Leveraging the tacit knowledge and lived experiences [39,40] of individuals from a community helps to understand how everyday tasks are conceptualized, approached, and completed. This process is typically made easier through in-person design sessions. The benefits of in-person collaboration include rapport building [41], a sense of ownership from participants who co-design the system [42], shared values on which design facilitators can build energy within the participatory design methodology [39], as well as perceiving subtle cues of interpersonal communications such as facial expressions and body language [40]. During in-person design sessions, brainstorming and drawing activities are conducted using tangible tools, including butcher paper (a type of heavy paper hung on walls to collect and record ideas) and sticky notes for rearranging the linkages, groupings, and prioritization of specific ideas, allowing for rapid iteration. These activities, and their necessary physical tools, support collaboration and inspire meaningful dialogue between participants and researchers [43].

Approaches to evaluating participatory design have focused on the processes deployed, effects on designers, and outcomes such as satisfaction and empowerment [44,45]. Evaluation of the design process and decision-making have included the collection of qualitative data from end users and system developers to determine the effectiveness of the decision-making [46]. When the focus was on the effect of the design process on participants, evaluators concentrated on participant experiences through interviews [44]. Outcomes, such as participant gains, can also be evaluated, by assessing participant experiences through interviews or surveys to measure how the design product benefited them and if that benefit lasted [47]. The methodological discipline of participatory design is based on meaningful communication between participants and researchers and allows decision-making by participants, such as how health information is accessed and displayed.

The TGHIR design process coincided with the early months of the COVID-19 pandemic, which forced a transition from in-person to web-based design sessions. Although remote collaboration for design-based user experience interviews was becoming more common [48,49], the use of a suite of web-based tools to support web-based design collaboration has been less documented, especially in the TGD community [50]. Early participatory design studies [50-52] focused on how communication technologies such as email and websites could organize the web-based design process. The effective use of these communication tools, in combination with a shared web-based space hosted on the internet where design could happen synchronously or asynchronously, led to the advancement of how participatory design could be implemented. The addition of shared web-based creative spaces supported participation among remote team members while requiring fewer resources.

Web-based participatory design to support community-driven development of products on an asynchronous discussion forum has proven to be successful. Research conducted by Hess and Pipek [53] indicated that engaging web-based communities to support community-driven development of consumer software is possible, especially if the work is intrinsically fun. The authors found that participants on the web could contribute to the design process of a software system. However, the project began to feel unpaid by some members. A power balance between participants on the web and professional designers was observed and influenced the decisions made for the system. The findings of this study suggested that the responsibilities of participants on the web should be limited to distinct use cases so the development process is not dominated by the most engaged volunteers who might have affordances, such as levels of experience or more time to participate, which may allow greater influence on design decisions. Our proposed web-based participatory design addressed some of these challenges.

Participatory design has been used by other research teams to support the design of health information resources [54-59]. This project was potentially the first to engage TGD participants exclusively in a web-based participatory design process. The web-based method of engagement might safeguard privacy and safety and allow greater involvement of TGD individuals in research. The overall objective of this paper was to describe our web-based participatory methodology and engagement evaluation for design session 1.

## Methods

### Overview

The setting was the University of Colorado Anschutz Medical Campus, an urban academic medical center, in collaboration with the University of Colorado Integrated Transgender Clinic, and One Colorado, the state’s leading advocacy group for LGBTQ (lesbian, gay, bisexual, transgender, and queer) persons. The design and development of the TGHIR were guided by a participatory process to ensure that the final TGHIR design would serve the health needs and goals of the TGD community. The process involved deploying a series of iterative methods to explore the use context and needs of end users. We described and characterized web-based methods and engagement, including qualitative insights from participants on the design of the TGHIR and the number of influential verbal and web-based participant interactions.

### Recruitment

There were 3 groups involved in the design of the TGHIR: researchers, advisers, and participants. Researchers were responsible for facilitating the design sessions and implementing the decisions made in partnership with the advisers and participants as well as the agile [60] development of the resource. Advisers were partners [61,62] in the design process and provided feedback on participant engagement strategies, insights from design sessions, and the development process. Participants were responsible for generating ideas and making decisions on how the TGHIR should be designed, features to include, and wireframing.

Participants had to be aged 18 years and meet one of the following two inclusion criteria: (1) self-identified as TGD at any point in their journey or (2) be parents or guardians of a TGD youth.

Recruitment took place during the COVID-19 stay-at-home orders from the State of Colorado. Strategies for recruitment included posting web-based flyers in TGD Facebook groups and delivery through listservs. The outreach was led by a local TGD community partner, One Colorado [63]. We conducted a thorough eligibility call over the Zoom videoconferencing platform (Zoom Video Communications) with each participant to assess their interest in transgender health and how they would like to contribute, which allowed the researcher to confirm the intent of their interest and that the participants could connect to Zoom [64].

Community Advisory Board members, known as advisers, were nominated, after funding for the project was obtained, by partners and allies in the transgender community, One Colorado, and the Integrated Clinic at the CU Anschutz Medical Campus. The process of adviser selection included a nomination phase and an interview phase to assess whether the nominee’s goals and motivation fit with the objectives of the project. All nominated individuals were onboarded. Community Advisory Board membership included 8 TGD individuals, 3 parents of TGD adolescents, 1 advocate from One Colorado, 4 health care providers who served the TGD population, 1 library scientist, and 4 research staff, totaling 18 members (3 advisers were in 2 of the categories described above). Advisers attended a 4-hour in-person kickoff meeting and eight 1-hour web-based meetings. The topics of the 8 web-based meetings included purpose-to-practice exercise, identifying credible health information, asynchronous work group planning, a discussion on the Black Lives Matter movement in the summer of 2020, a review of health care provider resources, focus group and design session data discussion, TGHIR app demo feedback, usability testing data discussion, and a celebration to acknowledge what we achieved together. Each adviser was compensated US $50 per hour for attending the meetings.

### Participatory Design Sessions

A web-based design approach required adapting in-person participatory design approaches to understand the targeted end users, the tasks end users were attempting to complete, and the environment in which participants completed the tasks [59]. The three stages of the TGHIR participatory design approach were adapted from Spinuzzi [65] and included (1) initial exploration of end-user needs, (2) discovery processes of prioritization and ideation on potential outcomes, and (3) prototyping. As a research team, we integrated these stages into a larger design process to access the opinions and experiences of participants on behalf of the targeted end users. A total of 4 distinct design sessions were conducted as part of the TGHIR design process (Table 1). We described the methodology implemented in these sessions, including how the methods were adapted to web-based interactions and the degree of participation in the “Ladder of Participation” column [61,62], a theoretical construct that described the range of co-designer participation from low to high.

Several web-based tools were used for web-based participatory design session implementation, including Zoom [64], Mural (Mural) [66], REDCap (Research Electronic Data Capture; Vanderbilt University) [67], and Justinmind (Justinmind) [68]. Zoom was used primarily to allow all participants to meet, with audio and video capabilities using a computer or tablet and computing functionality in a common web-based space. Mural was used as the collaborative workspace for the design sessions; participants were asked to create free accounts and were then sent a link to collaborate in a Mural workspace. REDCap was used for web-based surveys and Justinmind software was used for wireframing and prototyping.

**Table 1:** Four design sessions.

Design session and goals (research participants)	Participatory design stage adapted from Spinuzzi [65]	Ladder of participation	Web-based tools	Planned in-person tools
Session 1: Exploration of potential features (n=4)	Stage 1: initial exploration of end-user needs	Consultants: participant feedback on features	Mural and Zoom	Butcher paper, sticky notes, markers, and bullseye visualization
Session 2: Feature prioritization (n=22)	Stage 2: prioritization (Kano) and ideation on potential outcomes	Consultants or partnership: participant feedback via Kano and decisions on prioritization	Mural, Zoom, and REDCap^a^	Paper survey, butcher paper, sticky notes, markers, and bullseye visualization
Session 3: Iterative prototyping 3.1: Wireframing and prototyping (n=4) 3.2: Account creation (n=4) 3.3: Aesthetics(n=1)	Stage 3: prototyping	Partnership: participants decide how the resource displays each feature	Mural, Zoom, and Justinmind	Butcher paper, sticky notes, markers, PowerPoint to create first draft screen visuals, prototyping software
Session 4: Usability testing (n=2)	Heuristic evaluation: cognitive walkthrough	N/A^b^	Zoom, REDCap, and the TGHIR^c^	The TGHIR, paper, and pencil

^a^REDCap: Research Electronic Data Capture.

^b^N/A: not applicable.

^c^TGHIR: Transgender Health Information Resource.

### Design Session 1: Exploration of Potential Features

We described and characterized engagement in this session and provided findings in the results. Overall, design session 1 included 4 participants who identified as transgender (2/4, 50%) and nonbinary (2/4, 50%). The races reported by participants included American Indian (1/4, 25%), multiple races (1/4, 25%), and White (2/4, 50%). Age ranges were as follows: 20 to 29 years (1/4, 25%) and 30 to 39 years (3/4, 75%). Participants lived in rural areas (1/4, 25%) and urban locations (3/4, 75%).

**Figure 1:**
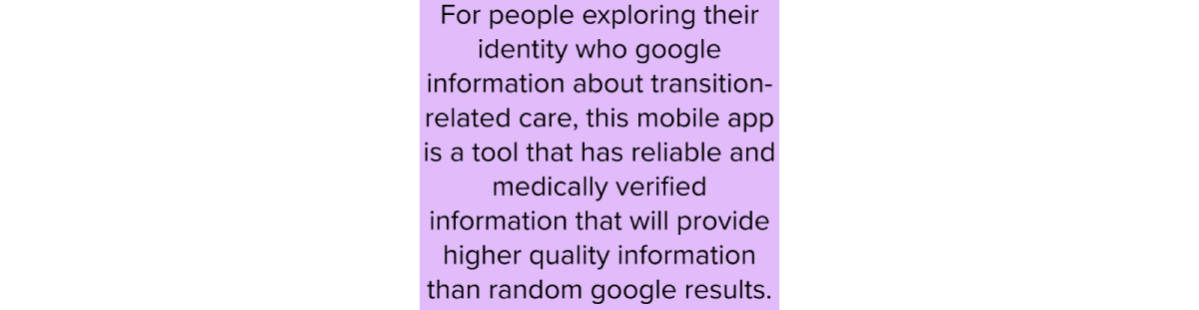
Persona statement.

Design session 1 was exploratory, informed by stage 1 of participatory design approach by Spinuzzi [65] and facilitated by the first author, a member of the research team. The focus was on gathering information from participants about the mobile resources end users in our target audience liked using and what features made their experience with the resource enjoyable. Participants developed personas through an exercise in which they ascribed feelings, values, and behaviors, resulting in potential use cases. An example of a persona statement is provided in Figure 1.

**Figure 2:**
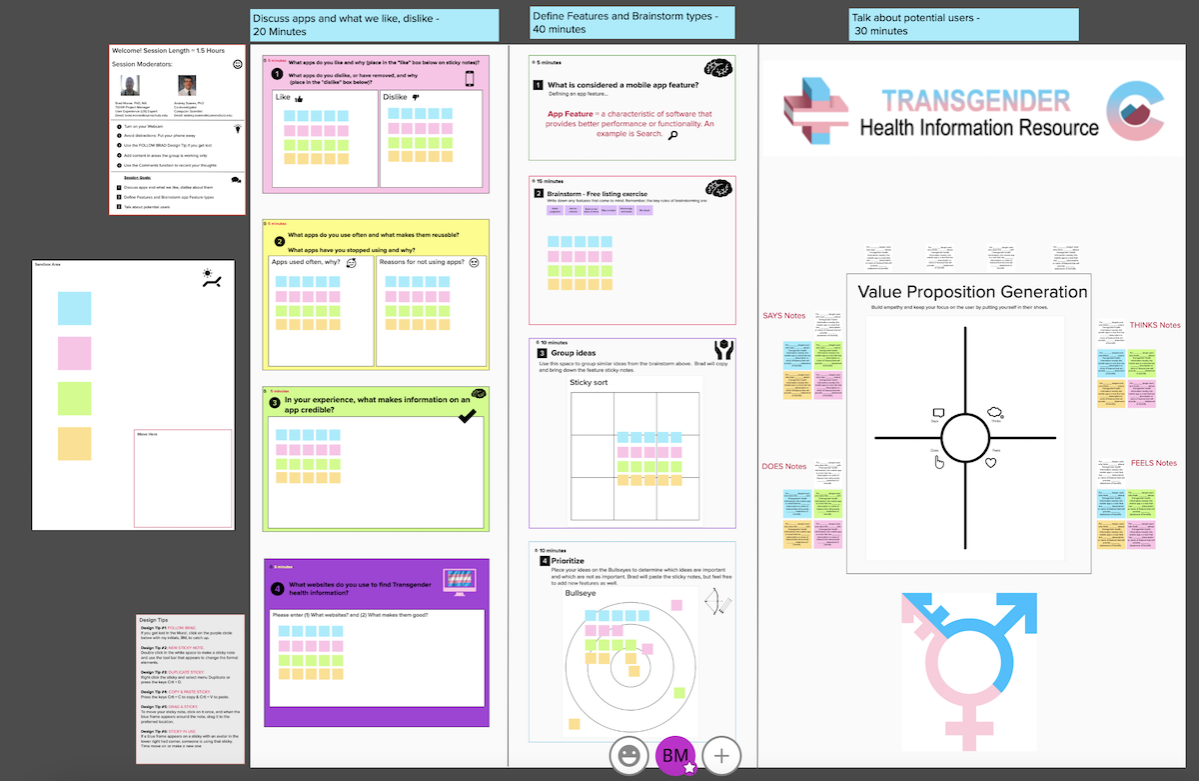
Mural collaborative space.

Mural (Figure 2), a web-based collaborative platform, allowed us to use the electronic counterparts of butcher paper, sticky notes, electronic pens with assorted color ink, and distinct colors and sizes of fonts.

We held a 10-minute Mural training session to optimize the time we had with the participants. Owing to the exploratory nature of design session 1, multiple brainstorming activities were conducted. Typically, participants wrote on sticky notes and organized their ideas on a whiteboard. Mural allowed for this functionality. In this session, we asked participants a series of questions about their mobile resource preferences, including, (1) What mobile apps do you like and why, (2) What mobile apps do you dislike and why, (3) What mobile apps do you use often and what makes them reusable, (4) What mobile apps have you stopped using and why, (5) What makes information on a mobile app credible, and (6) What websites do you use to find health information? Design session 1 also included an activity in which participants placed mobile resource features identified in the brainstorming activity on a bullseye image indicating how important the feature was to them.

### Design Session 2: Feature Prioritization

Design session 2 focused on discovering and prioritizing resource features identified in the first design session using stage 2, the discovery processes, of the participatory design approach [65]. The prioritization of features was organized using a Kano model of customer satisfaction [69-72]. This method assessed participants’ opinions regarding a feature being implemented [69]. The transition to web-based implementation required our design team to collect the Kano survey of customer satisfaction through REDCap instead of on paper and in-person. In this exercise, we used Mural to display the features generated by participants in design session 1 so the feature could be prioritized. The participants went through the survey as a group but responded individually.

**Figure 3:**
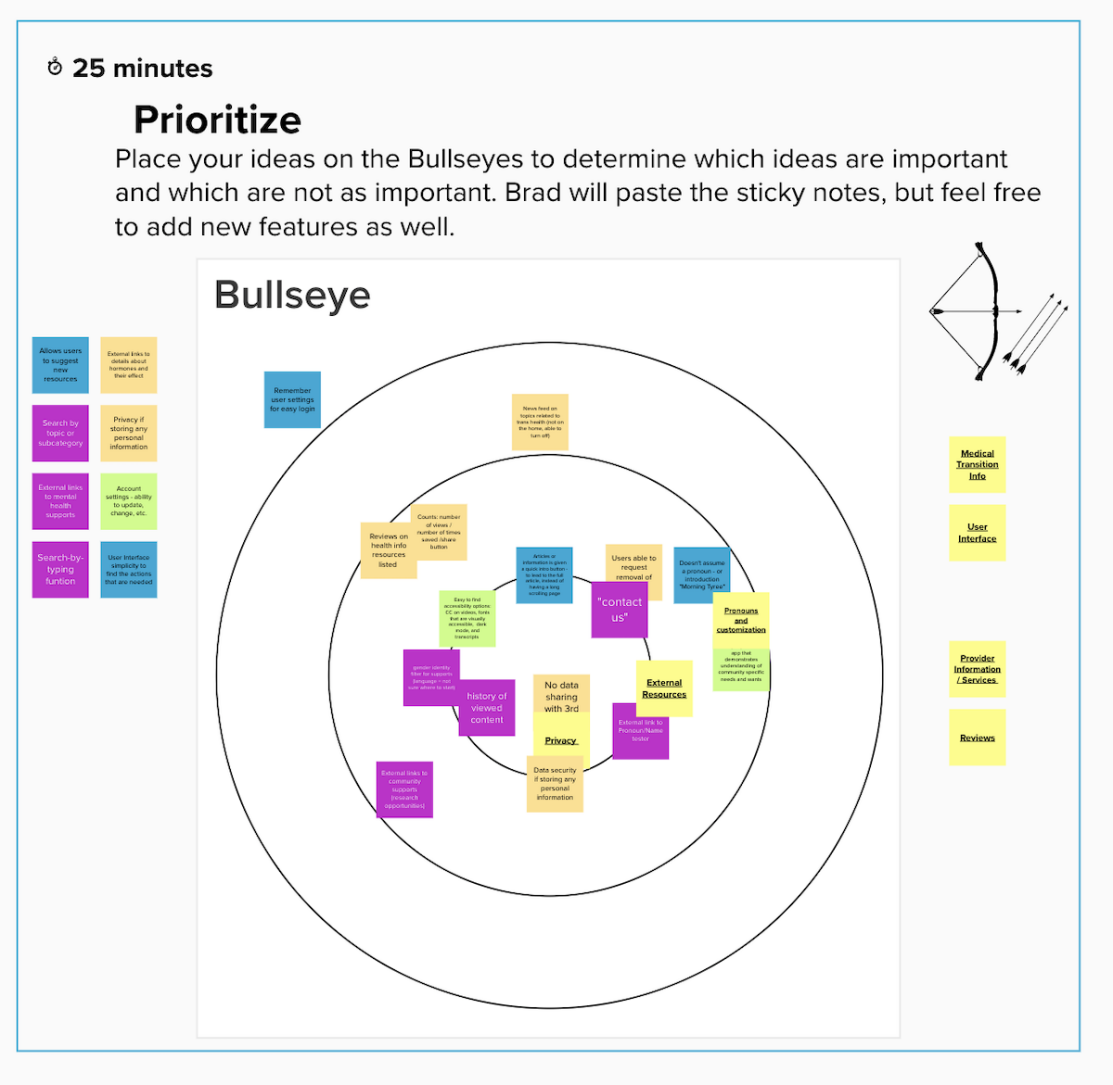
Bullseye design exercise.

After the Kano survey was completed, a second round of placing features and categories on a priority bullseye visualization (Figure 3) was conducted as a design exercise, furthering the discussion on what features were the most desirable to end users and should therefore be developed first. The design session 2 bullseye exercise was compared with how features were prioritized using the Kano survey. In addition, this exercise allowed participants to group the features into categories, such as in-person card sort, by grouping different sticky notes that referred to similar or the same type of features [73,74]. The last activity in design session 2 was the initial prototyping of the home menu and health resource preview, which was performed in Mural.

### Design Session 3: Iterative Prototyping

Design session 3.1 and design session 3.2 focused on wireframing, a process in which a sketch is made of what a product, in this case, a user interface, may look like. The wireframe was used as a starting point for the design work [75] and prototyping of the health information resource as an intentional and planned health information–seeking experience in stage 3 of the participatory design approach [65]. This work was performed using the Justinmind tool, a program that allows designers to rapidly create interfaces and modify them in real time. Using Justinmind, we created wireframes and asked participants for input on the design and implied functionality using Zoom. Participants first responded to the design elements of the baseline design created by the researchers. In traditional in-person sessions, the pencil and paper design methodology allowed for a quick iteration of the initial design during an in-person session. In this web-based design session, the use of a baseline design allowed the researcher to engage more quickly and in greater detail about the interface because time was not used to set up the initial design. Design session 3.3 was held with one participant to collect insights into aesthetics, or look and feel, of the TGHIR.

### Design Session 4: Usability Goal-Oriented

The fourth and final design session occurred after the TGHIR was built and consisted of a cognitive walkthrough heuristic evaluation, in which an experienced mobile health researcher and a behavioral scientist were asked to perform navigationally based tasks, assuming that most tasks embedded in the TGHIR would be goal-orientated [76], for example, find and like a resource. Participants installed the resource on their phones for an authentic experience.

In the usability testing sessions, Zoom, REDCap, and TGHIR were used. In our initial plans, this interaction would have been in-person and the participant would have had the TGHIR in their hands, as we observed their use of the resource and recorded the necessary usability data. Instead, design team members observed the participants through a Zoom connection. Usability evaluation tasks (Table 2) were described and completed by the participants [77,78]. These tasks were prioritized because they were associated with the features participants identified as important for the TGHIR to access credible health information.

**Table 2:** Evaluation tasks.

Task	Action to be completed
1	Create account and view consent and privacy language
2	Select preferred health information categories
3	Find a specific health information item using the category cards
4	Find and use the filter to narrow the resources to a relevant informational item
5	Like a resource
6	Bookmark a resource
7	Locate and use the search function to find a specific health information resource
8	Send a message to the developers
9	Share a new resource for the community with the developers
10	Find the most liked health information resource

### Data Analysis

We analyzed the video recording from design session 1 and the artifact created in the Mural collaborative space to describe and characterize web-based engagement. Design session 1 was recorded using the Zoom tool and transcribed by a professional transcriptionist. A research team member performed rapid analysis [79] of the transcriptions to quickly identify key points and comments that reflected participant engagement and major design decisions. Exemplar quotes were provided to highlight the impact of comments on the final design and the resulting features developed in the resource. Quantitative data included length of each design session exercise, number of verbal comments by all participants, total verbal comments, number of times the Zoom camera was turned off by participants, number of Mural interactions (creation or manipulation of digital Mural content) by participants, and total Mural interactions by design exercise. For a verbal comment or Mural interaction to be counted, the comment or interaction had to be considered influential in advancing the design process. Comments or interactions that expressed confirmation or agreement were not considered influential.

### Ethics Approval

Informed verbal consent was obtained from all participants. Each participant was paid US $50 for each of the 1.5-hour design sessions they attended. Project approval was obtained from the University of Colorado’s Human Research Ethics Committee, Colorado Multiple Institutional Board (COMIRB# 19-1562).

## Results

### Recruitment

Eligibility screening was performed using Zoom. A total of 41 individuals were screened for eligibility; of these, 27 (66%) were eligible and available to participate in at least one of the design sessions. Mural relied on well-established computing conventions to add, edit, and delete content and was, therefore, familiar to the participants who quickly mastered the skills to work alongside the research staff in the collaborative space.

### Design Session 1

In design session 1, participants identified the following specific health information for inclusion in the resource: affirming care (supportive care for the TGD community [7]), affordable medical options, information on transition, information on successful transitions (transitions were associated with the period during which a person began to live according to their gender identity rather than the gender they were thought to be at birth [80]), family resources, community support, and medically verified information.

Responses to the questions addressing participants’ mobile app preferences are presented in Table 3.

We provided the number of verbal comments and web-based interactions in design session 1 to show the volume of participant engagement in Table 4. Design session 1 was 119-minutes long; there was an average of 1.87 web-based points of engagement every minute. The researchers turned off their cameras to focus on the participants. Two participants experienced bandwidth trouble during the first exercise and chose to turn off their cameras to contribute to the web-based participatory design process. These 2 participants did not provide any other reason for their cameras being turned off and both continued to contribute despite the bandwidth problems they experienced.

Additional qualitative evidence for the effectiveness of the web-based participatory design process is presented in Table 5. Participant insights from the first design session remained prominent throughout the design process and directly affected the course of the design work. Participant’s verbal interactions were evident in the final TGHIR features.

**Table 3:** Questions addressing participant’s mobile resource feature preferences.

Question	Responses
What mobile apps do you like and why?	Gmail: simple design, easy to access, can limit notifications Facebook: easy to refresh Uber Reddit: the ability to search by subcategories Spotify Genius Scan Evernote: does a fair job of talk to text Merlin Bird ID: simple, easy to use, and regularly updated Mint: great UI^a^ and UX^b^ and never buggy Native Land: great multisource database and simple UI and UX AllTrails: surprisingly prefer using mobile over web app because of UI and UX
What mobile apps do you dislike and why?	Whole Foods: not intuitive Snapchat: too many notifications Your Turn: advertisements interrupting experience Tabletopia: optimized for PC and Tablet not mobile Spectrum Mobile: lack of control with certain items C25K: too many options Stitcher: too many options Apple Notes app: clunky and difficult to change formats Google sheets: difficult to use Apple Maps: location and information often totally inaccurate
What mobile apps do you use often and what makes them reusable?	Hearthstone: rewards frequent use Facebook: allows me to keep connected Reddit: plenty of media available Discord: able to access through multiple mediums Smarthub: saves relevant data for later Keep Notes: does exactly what it needs Instagram: addictive, friends, distraction Stitcher: do not like the interface, I just like the content, streaming, and downloading NYTimes: good usability Gmail: necessity and like UX better than other mail apps Spotify: seamless desktop to mobile transition
Reasons for not using an app	Excessive or random crashes Too many ads Excessive notifications Difficult to navigate; no search function Too many options Need to create an account when not necessary Ugly or outdated Uses too much power or memory on phone Busy user interface
What makes information on a mobile app credible?	Citing sources Current information Customer service Association with credible groups Not asking to rate the app Easy to navigate Inclusive language Transparency on who developed app
What websites do you use to find health information?	Private Facebook groups Forums like Reddit WebMD Denver Health PFLAG One Colorado Queer Asterisk

^a^UI: user interface.

^b^UX: user experience.

**Table 4:** Design session 1 engagement evaluation (total interactions, N=301).

Mural exercise	Exercise time (length of discussion)	Verbal comments by participants, n				Verbal comments per exercise, n	Web-based interactions by participants, n					Web-based interactions per exercise, n
		A^a^	B^a^	C^a^	D^a^		A^a^	B^a^	C^a^	D^a^		
What apps do you like	6:10-14:50	2	2	0	1	5	8	10	3	7	28	
What apps do you use	14:51-22:29	1	1	3	3	8	11	10	7	10	38	
Credible information	22:30-30:30	5	3	0	1	9	5	5	4	4	18	
TGD^b^ information	30:31-39:05	1	2	0	0	3	5	4	4	2	15	
Brainstorm—free listing	39:06-1:00:38	8	7	1	3	19	10	8	9	8	35	
Card sort	1:00:39-1:13:24	3	5	2	2	12	12	14	10	10	46	
Bullseye prioritization	1:13:25-1:28:00	7	7	3	3	20	8	12	8	0	28	
Value proposition generation	1:34:05-1:55:10	2	1	0	0	3	4	4	2	4	14	
Total	1:58:55 (including breaks)	29	28	9	13	79	63	67	47	45	222	

^a^Participants.

^b^TGD: transgender and gender-diverse.

**Table 5:** Exemplar quotes, impact, and design influence.

Session Exercise	Result		
	Comment	Impact on design	Influence on design
1.3	“I think having a channel in which folks can reach out if there’s an issue with information on the app...message customer support, call customer support or some kind of way that it’s not just, you know, you’re not searching really, you know, a long way through the app or trying to find a human to connect with within even an issue or a question”	Customer support as a priority within the app—visible, easily accessible. Important to make users feel valued and heard.	Contact Us page with the option of sending different types of messages to the TGHIR^a^ development team.
1.5	“I think it would be good if there is a place for it [the resource] to remember your name but also not to make it too cumbersome should you decide to change it. If it remembers your names also having an option for pronouns, even if it doesn’t pop up anywhere, it’s nice to have that affirmation.”	Function to remember names and customize pronouns within the resource. Demonstrates understanding of target audience.	Providing pronouns was eventually scrapped to avoid collecting data the resource would not use. This is the settings menu based on the comment above. The design attempts to make changing settings easy.
1.5	“My Verizon [app] can update some things on your account, but otherwise it’s just a giant ad for you to upgrade your system. I think interactivity, there’s a reason why you’ve downloaded an app instead of going to the website.”	Information should be presented in a mobile app that fosters easy access with an intuitive interactive navigation so the app could be used at any time and in any place.	The TGHIR was design and developed for iOS and Android, the 2 most common mobile app operating systems.
1.6	“I have the [Name], My Chart open. And one of the options is you can personalize it. The personalization is just changing the color for the frame of the app. That seems pointless to me...yeah, it just seems like such a superfluous option. I would rather move the icons around on the homepage...there might be something that I like to check more than other people. Moving the menu around would be a nice personalization. But changing the color is not worth it.”	If the resource is going to include customization options, it should go beyond the ability to simply change the colors on the interface, eg, the option to move icons around based on relevancy to the individual user. The orange cards are categories of interest selected by the user.	The Health Resource Cards could be pinned to the top of the page making them easier to return to during repeated use. Once the resource was pinned, the TGHIR app changes the card from blue to orange.

^a^TGHIR: Transgender Health Information Resource.

## Discussion

### Principal Findings

We found that using a web-based suite of collaborative tools with participants, we were able to effectively engage in productive discussions and make design decisions for the development of TGHIR. This approach to design work, in our case, was seamless and did not limit participants from engaging and providing meaningful influence on the resulting health information resources. The value of web-based recruitment and design sessions should be underscored because of the relative social safety of TGD participants. This recruitment method may be a strength for engagement and have a positive impact on TGD involvement in research by creating safe web-based spaces for TGD involvement.

We observed consistent engagement throughout design session 1. Upon reflection and analysis of the data, we found that the immediacy of engagement was impressive. It did not take participants much time to get involved and discuss design issues with their fellow participants. The dual interaction of manipulating web-based content in the Mural document and discussing the topic at hand may have led to a more immediate collaboration.

This study shed light on a web-based methodological approach to co-design health information resources within TGD communities. Through their involvement and enthusiasm for the work, participants indicated that a web-based approach to design was appropriate and can be used instead of resource-intensive in-person gatherings. Queer and trans communities have embraced digital technologies in radically affirming ways [81,82] to move beyond the acceptance of unsatisfactory options. The uptake of web-based design, and the necessary digital technologies, in TGD communities is feasible.

Mural interactions indicated that participants were consistently present throughout the design process. With 222 Mural interactions across 4 participants, it was evident that these individuals were highly engaged on the process. Whether this level of engagement can be replicated or generalized to other communities and topics requires further research. The verbal comments provided evidence that the interactions were process-oriented and moved the discussion to inform the development of TGHIR. Furthermore, specific design decisions were made owing to the input of the participants, providing evidence of their importance in the web-based design process and speaking to the effectiveness of web-based participatory design.

### Strengths, Limitations, and Future Directions

The strength of this project was the introduction of a method to describe and characterize engagement and interactions during participatory design facilitated by web-based means. The first limitation was that this study evaluated the engagement of participants from the TGD community but did not empirically compare the quality or quantity of engagement with an in-person design process. Future research should examine in-person design sessions compared with web-based design sessions to evaluate and compare the quality and quantity of engagement and interactions. Second, our recruitment method used Facebook to identify participants. This approach yielded a predominantly educated, professional, urban, and white sample. Although we recruited enough TGD individuals for the design of the TGHIR, we acknowledge that this sample may not have been representative of the overall population. As a result, we sought and were awarded funding to test the resources using a diverse research sample. This study is currently underway. Third, the small number of participants was a limitation. In future work, more participants should be involved in the participatory design process to determine whether high levels of web-based participation are maintained in larger samples. Finally, although web-based means of participation helped during the COVID-19 pandemic, a limitation was that it might have also been difficult to reach individuals who did not have the means or ability to access the requisite resources for web-based collaboration, such as high-speed internet and private space [83]. It will be important to keep this accessibility issue in mind when recruiting participants.

### Conclusions

Our results had important implications for the use of web-based methodologies in the design of health information resources. Web-based participatory design can support opportunities to contribute despite the potential logistical barriers of in-person design sessions by offering multiple convenient design session times and multiple interaction options. In addition, this approach is helpful when recruiting members from marginalized communities that are small and geographically dispersed, especially rural communities. Not only does the web-based methodological approach work during a pandemic but it may also help when there is historic distrust of research and health care from a community that has been stigmatized and experienced discrimination, such as the TGD community, by researchers and medical providers.

Our evaluation of the web-based participatory design indicated that web-based design sessions can engage participants in creating satisfactory interfaces for accessing and consuming health and medical information. Obtaining web-based input from participants was possible and efficient. Web-based recruitment is also possible for individuals who belong to marginalized communities and provides a platform through which these individuals can safely communicate with others in their community to design health information resources. Integrating web-based platforms can effectively engage participants and yield a positive user experience. Multiple participants reported that a health information resource of this nature would have been helpful in their journey toward gender identity exploration or gender transition.

## Abbreviations

LGBTQ: lesbian, gay, bisexual, transgender, and queer
REDCap: Research Electronic Data Capture
TGD: transgender and gender-diverse
TGHIR: Transgender Health Information Resource

## Data Availability

Relevant data associated with, created, and used for the evaluation presented in this paper are openly available from the University of Colorado Strauss Health Sciences Library, Anschutz Medical Campus, Data Repository [84].
